# Differential Inhibition of *Helicoverpa armigera* (Hubner) Gut Proteinases by Proteinase Inhibitors of Okra and It's Wild Relatives

**DOI:** 10.5402/2013/632173

**Published:** 2013-02-03

**Authors:** Shilpa K. Udamale, M. P. Moharil, T. B. Ugale, J. M. Mankar

**Affiliations:** Biotechnology Centre, Dr. Panjabrao Deshmukh Agricultural University, Akola, Maharashtra, India

## Abstract

The seeds of ten genotypes and twenty-nine wild relatives of okra were analysed for the presence of trypsin, chymotrypsin, and *Helicoverpa* gut proteinases (HGPs) inhibitors (HGPIs), with the aim to identify potent inhibitors of *H. armigera* gut proteinases. Proteinase inhibitors (PIs) obtained from wild relatives of okra exhibited stronger inhibition of HGPs than the genotypes of okra. In *in vitro* inhibitory assay against HGPs, *A. tuberculatus* 90396 and 90515 showed high tryptic inhibitory (71.8% and 69.2%), chymotryptic inhibitory (68.5% and 66.2%), and *Helicoverpa* gut proteinase activity (70.2% and 68.2%). In electrophoretic profile showed the same variation in the number of trypsin inhibitors (TIs), chymotrypsin Inhibitors (CIs), and HGPIs isoforms with different intensities, whereas genotypes of okra mostly showed monomorphic profile. Maximum eight HGPIs isoforms were found in *A. tuberculatus* (90396 and 90515). In bioassay studies, significant reduction in weight of *H. armigera* larvae was found, when larvae fed on PIs obtained from *A. tuberculatus* (90396 and 90515). Thus, the result of the present investigation indicates that further exploration of PIs obtained from *A. tuberculatus* (90396 and 90515) will be helpful for developing PIs-based insect resistance management strategies.

## 1. Introduction

*Helicoverpa armigera*, Hubner (Lepidoptera: Noctuidae), a highly devastating, polyphagous crop pest, has a broad host spectrum and causes a significant yield losses in many agriculturally important crops, like cotton, chickpea, pigeon pea, corn, maize, tomato, okra, sorghum, pearl millet, sunflower, and groundnut [1]. Thirty percent of all pesticides used worldwide are directed against *H. armigera* which resulted in high levels of insecticide resistance in this pest. Insecticide resistance in *H. armigera* is widespread problem in India, Pakistan, China, Australia, Thailand, and Indonesia [2]. The use of *Bacillus thuringiensis* (Bt) either in the form of formulation and transgenic plant may lead to develop resistance in insect in a short period of time, since many insect pests have developed resistance to Bt-like chemical pesticides [3]. Therefore, it is important to search and develop alternative methods of controlling this pest and proteinase inhibitors (PIs), constituents of natural plant defense system, promises to lead in this aspect in near future [4].

 Plant synthesizes various proteinaceous compounds against an insect attack, among the several plant defense proteins. PIs are abundantly present in seeds and storage tissues which represent up to 10% of the total protein [5]. PIs act as antimetabolic proteins, which interfere with the digestive process of insects. PIs are particularly effective against phytophagous insects and microorganisms. The defensive capabilities of PIs rely on inhibition of proteinases present in insect guts or secreted by microorganisms, causing a reduction in the availability of amino acids necessary for their growth and development. Most PIs interact with their target proteinases by contact with the active (catalytic) site of the proteinase resulting in the formation of a stable proteinase-inhibitor complex that is incapable of enzymatic activity [6].

 Preliminary studies on the presence of proteinase inhibitors from seeds of okra by Ogata et al. [7] found that PIs from okra inhibited both bovine trypsin and chymotrypsin, which are typical digestive enzymes. This study showed that okra seeds contain PIs of trypsin and chymotrypsin which constitute the defense machinery.

 In the present work, we have screened several okra genotypes and its wild relatives for the presence of PIs, and we have identified several potent and high potential PIs in wild relatives of okra. Bioassays were performed to ascertain the potency of the okra inhibitors in inhibiting the growth of *H. armigera* larvae. This information can be exploited for planning the strategies for developing insect resistance transgenic plants in the future.

## 2. Material and Methods

### 2.1. Seed Material and PI Extraction

 Seeds of the different genotypes of okra and its wild relatives used in the present investigation are detailed in Table 1.

Cultivated genotypes of okra were kindly provided by a senior research scientist from the Chilli and Vegetable Research Unit, Dr. P.D.K.V., Akola, India, and wild relatives were obtained from NBPGR, India. 

 Dry seeds were grounded to a fine powder, defatted, and depigmented with six washes of acetone and hexane. The solvent was filtered off and the seed powder was obtained upon air drying. The powders were mixed with five volumes of 0.1 M sodium phosphate buffer (SPB), pH 6.8, and kept overnight at 4°C for extraction with intermittent shaking. The suspension was centrifuged at 12,000 rpm for 20 min at 4°C and the supernatant was stored in aliquots at −20°C. The protein content of the extract was determined by Bradford's method [8]. 

### 2.2. Extraction of HGPs

 The late third or the early fourth instar larvae, from homogenous culture of *H. armigera*, were dissected and the midgut was isolated and stored frozen at −78°C. Required gut tissue was homogenized in 1 volume of 0.2 M glycine-NaOH buffer, pH 10.0, and kept for 2 h at 10°C. The suspension was centrifuged at 12,000 rpm for 20 min and the supernatant was used as a source of HGPs.

### 2.3. Electrophoretic Visualization Of HGPs

 HGPs were detected by using SDS-polyacrylamide gel. Enzyme was extracted (20 *μ*L) from the midgut of *H. armigera* larvae and electrophoresed on 12% SDS-polyacrylamide gels along with treatment buffer 60 mm Tris-HCl, pH 6.8, 2% SDS, 20% glycerol, and 0.1% bromophenol blue [9]. After electrophoresis, SDS-polyacrylamide gel was washed in 2.5% Triton X-100 for 10 min to remove SDS, then incubated in 2% casein in glycine-NaOH,  pH 10, and the gel was then stained with coomassie brilliant blue R-250. HGPs bands were revealed as white bands with dark blue background.

### 2.4. Proteinase and PI Assays

 Total proteinase activity was measured by azo-caceinolytic assay [10]. For azo-caceinolytic assay, midgut homogenate was mixed with (130 *μ*L) of Tris-HCl buffer, pH 9. To the above mixture, 100 *μ*L of 2% azocasein was added and incubated for 1 hr at 37°C. The reaction was stopped by adding 500 *μ*L of 5% ice-cold trichloroacetic acid (TCA). After centrifugation at 14000 rpm for 15 min at 4°C, an equal volume of 1 M NaOH was added to the supernatant and absorbance was measured at 420 nm. The protease activity of the sample was calculated using trypsin standard curve in terms of tryptic unit (TU).

 Tryptic and chymotryptic activities were estimated using the chromogenic substrates N-*α*-Benzyl-L-argine *p*-nitroanilide (BA*p*NA, Sigma) and N-Succinyl-Ala-Ala-Pro-Leu-p-nitroanilide (SAApLNa, Sigma) dissolved in dimethyl sulfoxide. Midgut supernatants were diluted 1 : 100 in buffer containing 100 mg/mL (200 mm Tris, pH-8.0, 20 mm CaCl_2_), 50 *μ*L were added to a microplate well, 50 *μ*L BA*p*NA for tryptic and SAApLNa for chymotryptic were added after 30 second incubation at 37°C, and absorbance was estimated at 405 nm.

 For the inhibitory assays, a suitable amount of inhibitor (30 *μ*L) and HGPs extract (50 *μ*L) was preincubated for 30 min at 37°C prior to the addition of substrate. *H. armigera* trypsin, chymotrypsin, and total gut proteinase inhibitory activities were estimated by using substrate BA*p*NA, SAApLNa, and azocasin. A total of 30 *μ*L proteinase inhibitor and 50 *μ*L gut extract were preincubated for 30 min at 37°C. After that, 50 *μ*L substrate were added to each well after 1 min incubation at 37°C, the reaction was terminated by addition of 500 *μ*L of 5% TCA, and absorbance was monitored at 405 nm. For total gut proteinase inhibitory activity, after adding 5% TCA centrifuged it and 50 *μ*L of 1 N NaOH were added and absorbance was estimated at 405 nm. One proteinase unit was defined as the amount of enzyme that increases absorbance by 1 OD/min and one PI unit was defined as the amount of inhibitor that causes inhibition of 1 unit of proteinase activity under the given assay conditions.

### 2.5. Electrophoretic Visualization of TIs, CIs, and HGPIs Isoforms

 TIs, CIs, and HGPIs isoforms, having the same amount of total soluble protein, were detected by using 10% polyacrylamide gel having 1% gelatin [11]. For TIs and CIs activity and in 0.2 M Gly-NaOH, pH 10, for HGPIs activity. The respective gels were transferred to solutions containing 0.1% trypsin or 0.1% chymotrypsin or HGP extract of equivalent activity and incubated for 1 hr with constant shaking. The gels were washed with warm water, fixed in 10% TCA, stained with Coomassie Brilliant Blue R-250, and destained. Isoforms were revealed as blue bands against white background.

### 2.6. Bioassay of PIs against *H. armigera* Larvae

 Bioassay was carried out at insect rearing facility of the Department of Entomology, Dr. P.D.K.V., India. Eggs, neonate, and early instars larvae of *H. armigera* were collected from the experimental field of Dr. P.D.K.V., India. This culture was maintained in the laboratory at 27°C at 80% relative humidity on fresh and soft seeds of pigeon pea until further use. Bioassay was carried out according to the protocol given by Bhavani et al. [12]. Fresh and soft seeds of pigeon pea were pressed by thumb and forefinger gently and put into multiwell rearing tray for releasing larvae. PIs from *A. tuberculatus* 90396 and *A. tuberculatus* 90515 (50 *μ*g of protein concentration) were loaded between the cavity of two crushed grains with the help of micropipette. Second larval instar of *H. armigera* was selected to start bioassay. Constant exposure of PI was maintained during whole experiment up to pupation of larvae.

 The observations of larval weights were taken after every 24 hrs after ingestion of food. Control population was also maintained simultaneously without PIs. The observation on larval mortality, larval weight, pupal weight, and number of malformed pupae and malformed adults were also recorded. CRD design was used for statistical analysis.

## 3. Results 

### 3.1. Activity and Visualization of Gut Proteinases of *H. armigera*

 Total gut proteinase (azocaseinase), trypsin-like proteinases (BApNAase), and chymotrypsin-like proteinases (SAApLNase) activities present in gut of *H. armigera* were assayed (Table 2). Total proteinases activity was observed to be 2.15 U/gut, and tryptic activity was found to be slightly higher (1.97 U/gut) than chymotryptic activity (1.87 U/gut).

**Figure 1:**
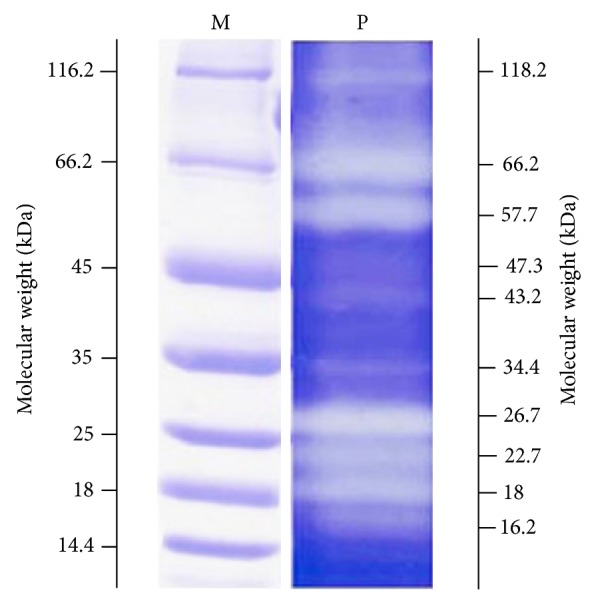
Isoforms of *Helicoverpa armigera *gut proteinases. M: standard molecular weight maker for protein. P: *Helicoverpa armigera* gut proteinases.

 Electrophoretic visualization of *H. armigera* gut proteinase isoforms was also carried out by SDS-PAGE, which is described in Section 2 (Figure 1). As revealed from plate 1, total *H. armigera* gut proteinase activity was distributed in ten isoforms, ranging from molecular weight 118.0 kDa to 16.2 kDa. The apparent density of P_1_, P_2_. 

### 3.2. Electrophoretic Profiles of TIs, CIs, and HGPIs Isoforms from Different Genotypes of Okra and Its Wild Relatives

PIs were isolated from ten genotypes of okra and 29 wild relatives used for electrophoretic visualization. Tis, CIs, and HGPIs isoforms, having the same amount of total soluble protein, were detected by using 10% polyacrylamide gel having 1% gelatin [11].

All wild relatives of okra showed variability in terms of the number and intensities of TIs bands. *A. tuberculatus* 90396 and 90515 exhibited the highest (six) TIs isoforms, and *A. angulossus* (203832) showed four TIs isoforms, whereas *A. ficulneus* (41748, 141042, 210361, and 140947) and *A. tetraphyllus* (90404) exhibited the minimum (one) TIs isoforms. All okra genotypes showed monomorphoic PIs profile, that is, four TIs isoforms were detected in all genotypes of okra with dark intensity, except Arka bahar which showed less intense TIs isoforms. 

Similarly, gelatin copolymerized polyacrylamide gel electrophoresis showed wide range of CIs (molecular weight 25.1 kDa to 6.3 kDa) with variable intensities. *A. tuberculatus* (90396, 90515, 90400, 140957 and 90402) reported maximum (five) CIs isoforms while *A. ficulneus* (140986, 141042, 210361, and 140947), *A. tetraphyllus* (92503), *A. moschatus* (141065), and *A. manihot* (329394) exhibited only one CIs isoform. Different okra genotypes exhibited maximum number of (four) of CIs isoforms, except Arka bahar which showed only one CIs isoform. Results clearly indicate the potentiality of *A. tuberculatus*, to search for new and potent proteinase inhibitors. This is also confirmed by our studies on TIs and HGPIs isoform.

**Figure 2:**
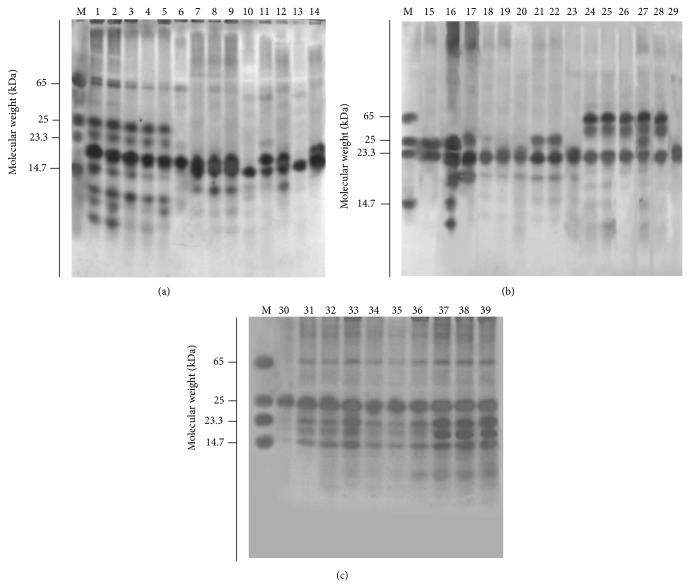
*Helicoverpa* gut proteinase inhibitors (HGPIs) isoforms from different genotypes and wild relatives of okra. (1).*A. tuberculatus* 90396, (2) *A. tuberculatus* 90515, (3) *A. tuberculatus* 90400, (4) *A. tuberculatus* 140957, (5) *A. tuberculatus* 90402, (6) *A. ficulneus* 140986, (7) *A. tetraphyllus* 90398, (8) *A. tetraphyllus* 90461, (9) *A. tetraphyllus* 90386, (10) *A. ficulneus* 41748, (11) *A. ficulneus* 141042, (12) *A. tetraphyllus* 92503, (13) *A. ficulneus* 210361, (14) *A. tetraphyllus* 90404, (15) *A. ficulneus* 140947, (16) *A. angulossus* 203832, (17) *A. angulossus* 203863, (18) *A. angulossus* 470751, (19) *A. manihot* 141019, (20) *A. manihot* 141045, (21) *A. angulossus* 203833, (22) *A. angulossus* 203834, (23) *A. manihot* 141012, (24) A. moschatus 140985, (25) *A. moschatus* 141056, (26) *A. moschatus* 141065, (27) *A. moschatus* 470737, (28) *A. moschatus* 470747, (29) *A. manihot* 329394, (30) Arka bahar. (31) Parbhani kranti, (32) AKO -107, (33) Arka anamika, (34) AKO-37, (35. Pusa A-4, (36) AKO-111, (37) AKO-102, (38) Adunika, (39) VRO-3, M: standard molecular weight marker.

To determine specificities of PIs towards HGP isoforms, PIs extracts were resolved on gelatin-polyacrylamide gel. Further, it was incubated with HGP extract obtained from midgut of *Helicoverpa* larvae (equal TI units), and HGPI bands were visualized as described in Section 2. Figures 2(a), 2(b), and 2(c) represent the electrophoretic profile of HGPIs in seed extracts of okra and its wild relatives. *A. tuberculatus* group showed presence of high activity HGPIs bands as compared to okra and other wild relatives. 

Among the wild relatives of okra, *A. tuberculatus* (90396 and 90515) showed eight HGPIs isoforms, whereas *A. tuberculatus* (90402) exhibited seven HGPIs band followed by *A. tuberculatus* (90400 and 140957) which showed six HGPIs isoforms and five HGPIs isoforms were found to be in *A. angulossus* (203832), whereas *A. ficulneus* (41748 and 210361) and *A. manihot* (329394) showed only one HGPIs isoforms (Figures 2(a) and 2(b)). Different genotypes of okra showed variable numbers of HGPI isoforms with different intensities. AKO-111, AKO-102, Adunika, and VRO-3 reported maximum (five) HGPIs isoforms with high intensity. Also Parbhani Kranti AKO-107, Arka anamika, and AKO-37 possessed four HGPIs isoforms, whereas Arka bahar consists only one HGPIs isoforms (Figure 2(c)). These results clearly showed that PIs from wild relatives of okra *A. tuberculatus* (90396 and 90515) exhibited strong inhibitory potential against HGP.

### 3.3. Inhibitory Potential of PIs from Different Genotypes of Okra and Its Wild Relatives against *Helicoverpa* Gut Proteinases 

 Several genotypes of okra and its wild relatives were analyzed for their inhibitory potential against HGP activity. Inhibition capacity of okra PIs towards HGP was evaluated by *in vitro* microplate-adopted enzyme assays. Low concentration of proteinase inhibitors (30 *μ*g) was used to obtain inhibition of tryptic, chymotryptic, and total gut proteinase activity. Control was maintained without any PIs and its activity was considered as 100%.

*Helicoverpa* gut consists of both tryptic and chymotryptic activity. Tryptic activity was slightly higher than chymotryptic activity. Therefore, inhibitory potential of PIs towards trypsin as well as chymotryptic activity was considered to be useful potent PIs.

Table 3 summarizes the inhibitory potential of PIs obtained from various okra genotypes and its wild relatives against *Helicoverpa* tryptic activity, *Helicoverpa* chymotryptic activity, and total proteinase activity. A close examination of data revealed that different okra genotypes possessed tryptic inhibitory activity ranges from 50.5% (AKO-37) to 63.9% (VRO-3). Amongst different wild relatives of okra, minimum inhibitory potential (39.9%) of tryptic activity was observed in PIs of *A. tuberculatus* (141042) and maximum tryptic inhibitory potential (71.80%) was observed in PIs of *A. tuberculatus* (90396) followed by *A. tuberculatus* (90515) which was 69.2%. A similar trend of inhibition was observed in case of *Helicoverpa *gut chymotryptic activity and *Helicoverpa* gut total proteinase activity.

### 3.4. Effect of Okra PIs Obtained from *A. tuberculatus* (90396 and 90515) on Fitness Parameters of *H. armigera*

**Figure 3:**
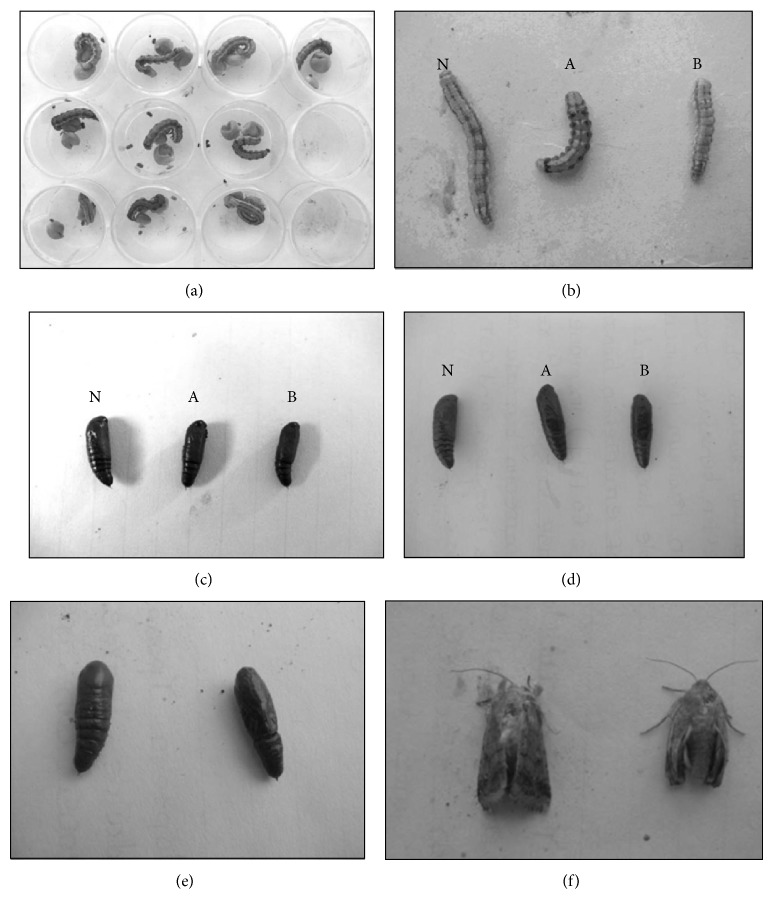
Effect of okra PIs on the growth and development of *H. armigera* larvae, whereas (A) larvae reared on PIs of *A. tuberculatus* (90396). (B) larvae reared on PIs of *A. tuberculatus* (90515). (N) larvae reared on artificial diet without PI. (a) *H. armigera* larvae fed on PIs obtained from *A. tuberculatus* 90396 and *A. tuberculatus* 90515, (b) larval weight reduction after feeding A and B PIs containing diet, (c) pupal weight reduction after feeding A and B PIs containing diet, (d) normal and malformed pupae, (e) normal pupae and dead pupae, (f) Normal and malformed adult.

 Bioassay results of PIs showed significant reduction in weight of *H. armigera* larvae when fed on PIs obtained from *A. tuberculatus* 90396 and 90515 (Tables 4 and 5, Figure 3). Also, effects on different parameters of *H. armigera* were recorded, namely, larval mortality, pupation rate, reduction in pupal weight, malformed pupae, pupal mortality, and malformed adult.

### 3.5. Day-Wise Reduction in Weight of *H. armigera* Larvae Feed with Okra PIs of *A. tuberculatus* (90396 and 90515) 

 The data (Table 4) on insect weight was affected by feeding with PIs obtained from *A. tuberculatus* (90396) and *A. tuberculatus* (90515), wild relatives of okra, indicating significant differences among the treatments. The wild relative *A. tuberculatus* (90396) was found to be the most effective. The mean of insect weight was 98.2 mg at 13 DAI, indicating a more significant reduction than the larvae fed on PIs whiuch was obtained from *A. tuberculatus* (90515) and artificial diet without PIs.

The second factor that is, age also showed significant differences indicating that the weight of the insect was directly proportional to the age of the insect. The interaction studies revealed that there was a significant reduction in insect body weight, when larvae fed with *A. tuberculatus* (90396) even at 12-13-day-old larva as well as pupal stage. 

### 3.6. Effect of Okra PI on the Growth and Development of *H. armigera*

 A total of 53.4% and 68.0% weight reduction was observed in larvae fed on *A. tuberculatus* (90396) and *A. tuberculatus* (90515) PIs containing diet (Figure 3(b)). Larval mortality was observed at 11 days after ingestion which on up to 40% in *A. tuberculatus* (90396) and 30% in *A. tuberculatus* (90515), whereas, in control, no larval mortality was recorded.

The larvae fed on proteinase inhibitor obtained from *A. tubercualtus* (90396) and *A. tubercualtus* (90515) forms blackish malformed pupae, in which the normal pupal were dark brown (Figure 3(d)). Pupation rate was lower in population fed on PIs of *A. tuberculatus* 90396 (60 %) followed by population fed on PIs of *A. tuberculatus* 90515 (70%) than that in the control. In addition, a significant decrease in pupal weight 54.1% and 68.5% was also observed in population fed on *A. tuberculatus* (90396 and 90515) as compared to the control (Figure 3). A total of 60% and 50% malformed pupae were found in population fed on PIs of *A. tuberculatus* (90396) and *A. tuberculatus* (90515), respectively, compared to the control, whereas pupal mortality was only 10% (Figures 3(d) and 3(e)). Okra PIs also exhibited adverse effect on adult emergence. After emergence, adults were found to be malformed (Figure 3(f)). 

## 4. Discussion

### 4.1. Activity and Visualization of Gut Proteinases of *H. armigera*

 Total gut proteinase activity in gut of *H. armigera* was found to be 2.15 U/gut. Tryptic activity was found to be slightly higher (1.97 U/gut) than the chymotryptic activity (1.87 U/gut). After visualization of gut proteinases, ten isoforms were observed, ranging from molecular weight 118.0 kDa to 16.2 kDa.

Earlier studies on proteolytic activity of lepidopteran insect gut showed that insect gut comprises of many isoforms of proteinases having diverse properties and specificities [13]. Harsulkar et al. studied the isoforms of gut proteinases of *H. armigera*, and their study revealed that *H. armigera* gut proteinase activity was distributed in six isoforms. Similarly, Potdar in 2008 studied proteinases of *H. armigera* gut, and he showed ten isoforms of proteinases in the gut of *H. armigera* [14]. 

 The presence of proteinases of different specificities in the midgut has a great significance for the survival and adaptation of phytophagous insects on several host plants. The adaptation of pests to host plant PIs probably results from the selection pressure acting on an entire insect population when they encounter PIs of their host plants [15]. Thus, ten isoforms of HGP found in present investigation supported the polyphagous nature of *Helicoverpa armigera*.

### 4.2. Electrophoretic Profiles of TIs, CIs, and HGPIs Isoforms from Different Genotypes of Okra and Its Wild Relatives

The TIs and CIs isoforms of wild relatives of okra showed significant variation with different intensity, whereas okra genotypes was exhibited monomorphic profile. A similar observation was also reported in pigeon pea by Chougule et al. [16], and they showed that pigeon pea cultivars exhibited monomorphism in TIs and CIs isoforms and diverse proteinase inhibitory profiles in pigeon pea wild relatives.

 Patankar et al. [17] also observed significant variation in the TIs isoforms from wild *Cicer* species. However, they have observed great conservation of TIs isoforms in the mature seeds of the chickpea cultivars. A similar observation exists in pigeon pea where TIs and chymotrypsin inhibitors are conserved in matured seeds of the cultivated pigeon pea, whereas a high level of diversity exist in uncultivated species of *Cajanus* [18, 19]. The variation observed in wild *Cicer* species is considered significant, as the TIs are known to serve as defense proteins against herbivores [20]. *Cicer reticulatum* and *Cicer arietinum* showed similar TIs band patterns, which suggests that *Cicer reticulatum* is genetically closer to *Cicer arietinum*. Thus, these studies can also be used for karyotyping the genotypes. The variation observed in the wild relatives of okra is considered significant, as TIs are known to serve as defense proteins against herbivores [20].

 Wild relatives of okra *A. tuberculatus* (90396 and 90515) showed eight HGPIs isoforms with high intensity, whereas, AKO-111, AKO-102, Adunika, and VRO-3 reported maximum (five) HGPIs isoforms, while Arka bahar consists only one HGPIs isoform (Figure 2). These results clearly showed that PIs from wild relatives of okra *A. tuberculatus* (90396 and 90515) exhibited strong inhibitory potential against HGP. Earlier studies on electrophoretic profiles of HGPIs of pigeonpea and its wild relatives. *Rhynchosia* group showed presence of high activity HGPIs bands (5) as compared to pigeonpea and other wild *Cajanus* species [16].

### 4.3. Inhibitory Potential of PIs from Different Genotypes of Okra and Its Wild Relatives against *Helicoverpa* Gut Proteinases 

 All results clearly demonstrate that okra PIs from *A. tuberculatus* (90396 and 90515) exhibited a stronger inhibition of HGP than cultivated genotypes of okra. Earlier studies on wild relatives of pigeonpea showed more than 70% inhibition, whereas, cultivars showed around 50% inhibition of HGP [16]. Moreover, the proteases from *H. armigera* were inhibited upto 85% by AKTI at a concentration 45 *μ*g mL^−1^ [21]. Previous studies showed that the *C. annum* PIs inhibited more than 60% total proteolytic activity [22]. A total of 72% total gut activity was inhibited by chickpea PI [15]. 

*H. armigera* is a polyphagous pest and possesses different types of proteinases in its gut [14], and the effectiveness of okra wild PIs offers good gene pool for the development of *H. armigera* (Bhendi fruit borer) resistant okra varieties; similarly, it offers good source to isolate PIs genes for developing insect resistance transgenic plants against *H. armigera*. 

### 4.4. Effect of Okra PIs Obtained from *A. tuberculatus* (90396 and 90515) on Fitness Parameters of *H. armigera*

 A total of 53.4% and 68.0% weight reduction was observed in larvae fed on *A. tuberculatus* (90396) and *A. tuberculatus* (90515) PIs containing diet (Figure 3(b)). Also larval mortality was observed up to 40% on *A. tuberculatus* (90396) and 30% on *A. tuberculatus* (90515), whereas in control no larval mortality was recorded. Pupation rate significantly decreases and 60% and 50% malformed pupae were found in population fed on PIs of *A. tuberculatus* (90396) and *A. tuberculatus* (90515). Okra PIs also exhibited adverse effect on adult emergence.

 The disruption of amino acid by the inhibition of protein digestion through PIs is the basis of PIs-based defense in plants; however, in nature it might be coupled with other factors. To evaluate *in vivo* effects of okra PIs on *H. armigera* feeding, assays were conducted with added inhibitor protein in the diet. Larval growth and development were dramatically reduced when larvae fed on okra PIs diet. Reduced feeding of larvae was observed in case of PIs incorporated diet than more that in the control, and the adverse effects were significant at a higher concentration of PIs doses. 

 Significant difference in larval mortality was also evident. This can be explained as the larval stage is very crucial for accumulating nutrients and energy, which is used for pupal and adult development. Starvation and added stress on gut proteinases expression system to synthesize new and higher amounts of proteinases could be the possible reason for arrested growth and mortality of *H. armigera* larvae. Other researchers also observed growth, retardation, and mortality with PI doses to *H. armigera* and other insects [12, 22–24]. Another interesting observation was that the inhibitor caused a high ratio of deformities in pupae and adult (Figures 3(d) and 3(f)), and such results were also shown by Franco et al. [25]. They reported 50% deformities in pupae and 81% in adult due to SKTI inhibitor. The requirement of lower PIs (50 *μ*L) in diet for maximum effect on *H. armigera* growth retardation indicates its high specificity towards HGPs.

 After extensive *in vivo* and *in vitro* screening of PIs from several cultivated and wild relatives of okra in the present study, PIs from *A. tuberculatus* (90396 and 90515) were found to possess potential, so as to explore it in the future for developing PIs-based management strategies of lepidopteron pest in general and *H. armigera* in particular.

## Figures and Tables

**Table 1:** Different okra genotypes and its wild relatives used in present investigation.

Serial Number	Germplasm

	Wild relatives of okra

1	*A. tuberculatus * 90396
2	*A. tuberculatus *90515
3	*A. tuberculatus * 90400
4	*A. tuberculatus * 140957
5	*A. tuberculatus *90402
6	*A. ficulneus *140986
7	*A. tetraphyllus * 90398
8	*A. tetraphyllus * 90461
9	*A. tetraphyllus *90386
10	*A. ficulneus* 41748
11	*A. ficulneus *141042
12	*A. tetraphyllus *92503
13	*A. ficulneus *210361
14	*A. tetraphyllus *90404
15	*A. ficulneus *140947
16	*A. angulosus *203832
17	*A. angulosus *203863
18	*A. angulosus *470751
19	*A. manihot *141019
20	*A. manihot *141045
21	*A. angulosus *203833
22	*A. angulosus* 203834
23	*A. manihot *141012
24	*A. moschatus *140985
25	*A. moschatus *141056
26	*A. moschatus *141065
27	*A. moschatus *470737
28	*A. moschatus *470747
29	*A. manihot *329394

	Cultivated varieties of okra

30	Arka bahar
31	Parbhani kranti
32	AKO -107
33	Arka anamika
34	AKO-37
35	Pusa A-4
36	AKO-111
37	AKO-102
38	Adunika
39	VRO-3

**Table 2:** *H. armigera* gut proteinases activity.

Serial Number	Proteinases	Enzyme activity (U/gut)
1	Total proteinase activity	2.15 ± 0.001
2	Tryptic activity	1.97 ± 0.003
3	Chymotryptic activity	1.84 ± 0.002

All the figures are mean of triplicate ± SE.

**Table 3:** *Helicoverpa* gut proteinase inhibitory potential of PIs isolated from okra genotypes and its wild relatives.

Serial Number	Genotype	HGP tryptic inhibitory activity (%)	HGP chymotryptic inhibitory activity (%)	HGP total proteinase inhibitory activity (%)

	Wild relatives of okra			

1	*A.tuberculatus *90396	71.8 ± 0.001	68.4 ± 0.004	70.2 ± 0.002
2	*A.tuberculatus *90515	69.2 ± 0.003	66.2 ± 0.004	68.3 ± 0.003
3	*A.tuberculatus *90400	62.4 ± 0.005	59.3 ± 0.005	61.4 ± 0.001
4	*A. tuberculatus *140957	67.0 ± 0.006	62.7 ± 0.003	60.6 ± 0.004
5	*A. tuberculatus *90402	60.4 ± 0.006	60.2 ± 0.004	62.2 ± 0.002
6	*A. fiulneus *140986	54.4 ± 0.005	50.2 ± 0.003	46.1 ± 0.002
7	*A. tetraphyllus *90398	49.4 ± 0.002	51.7 ± 0.003	43.1 ± 0.005
8	*A.tetraphyllus *90461	48.0 ± 0.001	50.5 ± 0.005	46.9 ± 0.002
9	*A. tetraphyllus *90386	51.2 ± 0.005	51.4 ± 0.005	45.0 ± 0.003
10	*A. fiulneus *41748	46.1 ± 0.002	44.1 ± 0.003	38.1 ± 0.003
11	*A. fiulneus *141042	39.9 ± 0.003	42.5 ± 0.003	42.7 ± 0.002
12	*A. tetraphyllus *92503	44.5 ± 0.001	41.8 ± 0.003	43.8 ± 0.002
13	*A. fiulneus *210361	47.0 ± 0.006	42.6 ± 0.002	40.5 ± 0.004
14	*A. tetraphyllus *90404	44.5 ± 0.004	43.3 ± 0.002	46.9 ± 0.003
15	*A. fiulneus *140947	43.4 ± 0.003	41.8 ± 0.002	43.5 ± 0.002
16	*A. angulosus *203832	65.3 ± 0.004	55.5 ± 0.006	59.1 ± 0.003
17	*A. angulosus *203863	53.0 ± 0.002	50.9 ± 0.003	46.1 ± 0.001
18	*A. angulosus *470751	50.2 ± 0.002	49.4 ± 0.003	47.3 ± 0.003
19	*A. manihot *141019	47.3 ± 0.002	48.7 ± 0.003	43.8 ± 0.002
20	*A. manihot *141045	42.4 ± 0.002	47.9 ± 0.001	42.9 ± 0.003
21	*A. angulosus *203833	51.5 ± 0.003	45.6 ± 0.003	51.5 ± 0.001
22	*A. angulosus *203834	48.0 ± 0.003	42.6 ± 0.004	46.5 ± 0.003
23	*A. manihot *141012	57.9 ± 0.002	45.2 ± 0.003	45.4 ± 0.003
24	*A. moschatus *140985	49.4 ± 0.001	49.8 ± 0.001	44.3 ± 0.002
25	*A. moschatus *141056	45.9 ± 0.003	44.1 ± 0.002	43.5 ± 0.002
26	*A. moschatus *141065	50.1 ± 0.002	51.7 ± 0.004	43.3 ± 0.003
27	*A. moschatus *470737	54.0 ± 0.003	54.7 ± 0.002	48.4 ± 0.003
28	*A. moschatus *470747	52.4 ± 0.004	52.0 ± 0.002	50.7 ± 0.003
29	*A. manihot *329394	42.7 ± 0.004	40.3 ± 0.002	41.9 ± 0.003

	Genotypes of okra			

30	Arka bahar	53.7 ± 0.004	46.0 ± 0.002	46.6 ± 0.002
31	Parbhani kranti	63.8 ± 0.005	62.1 ± 0.003	58.4 ± 0.004
32	AKO-107	53.9 ± 0.004	50.1 ± 0.003	56.8 ± 0.003
33	Arka anamika	55.1 ± 0.001	51.7 ± 0.003	52.6 ± 0.002
34	AKO-37	50.5 ± 0.004	48.6 ± 0.002	51.5 ± 0.001
35	Pusa A-4	51.9 ± 0.002	45.6 ± 0.004	54.5 ± 0.002
36	AKO-111	57.9 ± 0.003	50.9 ± 0.005	62.2 ± 0.003
37	AKO-102	65.6 ± 0.002	56.7 ± 0.003	60.3 ± 0.001
38	Adunika	60.0 ± 0.003	55.7 ± 0.001	63.3 ± 0.003
39	VRO-3	63.9 ± 0.002	61.9 ± 0.002	62.9 ± 0.002

**Table 4:** Day-wise reduction in weight of* H. armigera* larvae fed with okra PIs of *A. tuberculatus* (90396) and *A. tuberculatus *(90515).

Age (DAI)	Weight of larvae (mg) when fed with		
	A*. tuberculatus* (90396)	*A. tuberculatus *(90515)	Control (without PI)
1	23.8	25.3	27.3
2	28.2	31.0	31.7
3	38.3	40.0	43.3
4	47.1	50.1	55.0
5	54.7	56.7	72.3
6	71.0	89.7	98.7
7	91.0	120.3	138.0
8	116.0	130.0	162.7
9	121.3	141.3	186.7
10	127.6	156.0	221.3
11	144.7	180.0	259.7
12	156.3	211.7	297.7
13	176.3	224.7	330.3
Pupa	178.0	225.7	329.3

Mean	98.2	120.2	161.0

	Age	Variety	Interaction

F-test	Significant	Significant	Significant
SE	1.78	3.72	6.45
CD at 5%	4.96	10.32	17.88

Mean of all the survived larvae.

DAI: days after ingestion of proteinase inhibitor.

**Table 5:** Effect of okra PIs on the growth and development of *H. armigera*.

Growth and developmental parameters	Proteinase inhibitors	
	*A. tuberculatus* (90396)	*A. tuberculatus* (90515)
Larval mortality %	40	30
Larval wt. reduction % (control = 330.3 mg larval wt.)	53.4	68.0
Reduction in pupal wt. % (control 329.3 mg)	54.1	68.5
Malformed pupae %	60	50
Pupal mortality %	10	10
Malformed adult %	30	20
